# Human papillomavirus infection and cervical dysplasia in female sex workers in Northeast China: an observational study

**DOI:** 10.1186/s12889-015-2066-x

**Published:** 2015-07-23

**Authors:** Haiqing Jia, Xiaobin Wang, Zaiqiu Long, Liankun Li

**Affiliations:** Department of Gynecological Oncology, Liaoning Provincial Cancer Hospital, 44 Xiaoheyan Road, Shenyang, Liaoning 110042 China

**Keywords:** Female sex workers, Human papillomavirus (HPV), Cervical dysplasia, Risk factors, Shenyang, China

## Abstract

**Background:**

Women having multiple sex partners are reportedly at an increased risk of HPV infection. However, the prevalence and risk factors of HPV infection in female sex workers (FSWs) vary considerably across racial/ethnic, socioeconomic, and geographic groups. This study aimed to determine the prevalence and risk factors of HPV infection in FSWs in Northeast China.

**Methods:**

A total of 309 FSWs identified and approached through a local police office and 1000 healthy subjects from a single factor undergoing annual gynecological examinations in Shenyang were recruited. A liquid-based ThinPrep Pap test and the Hybrid Capture II-based high-risk HPV DNA test, with or without a colposcopic examination, were performed on both FSWs and control subjects. Data on HPV infection and histological and cytological lesions of the cervix were obtained and analyzed. A questionnaire survey was administered to all 309 FSWs with their socio-demographic and behavioral information collected. The association of various socio-demographic and behavioral variables with HPV infection was assessed.

**Results:**

HPV was significantly more prevalent in FSWs (61.90 %) than in healthy control subjects (21.00 %) (*P* < 0.01), so were cervical lesions (*P* < 0.01). HPV prevalence in our sample of FSWs fell in the upper range of reported values in FSWs across different countries, and was similar to that for FSWs in the southeast Chinese city of Huzhou but higher than that for FSWs in southwest China, Guangxi, as compared with data from other studies within China. HPV infection in FSWs was significantly associated with the age at first sexual intercourse (OR 0.699, 95 % CI 0.492–0.992) and post-menopause (OR 2.928, 95 % CI 1.099–7.800) (*P* < 0.05).

**Conclusions:**

FSWs are at a substantially high risk of HPV infection and cervical dysplasia development as compared with healthy control subjects in Shenyang, China. Age of first sexual intercourse and post-menopause are two independent risk factors for HPV infection in this special group of population. Intensified and coordinated efforts from government, public health sector, communities and families are needed to reduce the risk of HPV infection in this specific group of population.

## Background

Cervical cancer is one of the most common causes of cancer death in women, claiming more than 270,000 lives annually worldwide [1]. One of the most common types of cervical cancer is squamous cell carcinoma. Cervical dysplasia or cervical intraepithelial neoplasia (CIN) is the precursor of invasive squamous carcinoma of the cervix. Histologically, CIN is graded into mild dysplasia (CIN 1), moderate dysplasia (CIN 2), or severe dysplasia (CIN 3). Cytologically, cervical dysplasia is further categorized with the Bethesda system as: Atypical squamous cells of undetermined significance (ASC-US), atypical squamous cells – cannot exclude high-grade squamous intra-epithelial lesion HSIL (ASC-H), low grade squamous intraepithelial lesion (LGSIL or LSIL) consisting atypia and CIN 1, high grade squamous intraepithelial lesion (HGSIL or HSIL) primarily consisting of CIN 2–3 plus carcinoma, squamous cell carcinoma, atypical glandular cells not otherwise specified (AGC-NOS), atypical glandular cells suspicious for adenocarcinoma in situ (AIS) or cancer (AGC-neoplastic), and AIS. atypical squamous cells of undetermined significance (ASC-US), atypical squamous sells, Cannot Rule Out High-Grade Squamous Intra-epithelial Lesion (ASC-H) [2].

Genital infection with human papillomavirus (HPV), particularly the high-risk subtypes HPV-16 and HPV-18, is playing a major role in the etiopathogenesis of cervical cancer [3]; although the virus alone may not be sufficient to cause cancer [4], virtually all cervical cancers are associated with persistent infection with one of the high-risk types of HPV.

Since HPV is predominantly transmitted through sexual contact, HPV infection in women varies greatly with sexual activities and the number of sexual partners [5, 6]. In a recent comprehensive review paper, Soohoo et al. summarized data on the difference in HPV prevalence between female sex workers (FSWs) and the general population and on variations in HPV prevalence across different geographical regions of the world and showed that the median overall prevalence of HPV infection in FSWs in six WHO defined regions (i.e., Africa, Americas, Eastern Mediterranean, Europe, Southeast Asia and Western Pacific) was 42.7 %, significantly higher than that in the general population of women [7]. Moreover, these authors showed huge geographic variations (ranging from 2.3 to 100 %) in HPV prevalence in FSWs among the regions assessed. Nevertheless, of the 35 studies included in this review paper only one was from China, involving 810 female sex workers from Guangxi, a province in southwest China [8]. To date, no reports are available on the HPV infection and cervical dysplasia in FSWs in Shenyang, Liaoning, a province in Northeast China. Moreover, risk factors for HPV infection in FSWs in Shenyang have not been well defined, although the association of HPV infection with various demographic and behavioral variables has been assessed in FSWs in other studies [9–11].

This study was therefore conducted with two objectives: 1) to assess the prevalence of HPV infection and incidence of CIN in FSWs in Shenyang, China; and 2) to define putative risk factors for HPV infection in this unique group of population in Shenyang.

## Methods

### Ethical statement

The study was reviewed and approved by the Institutional Review Board of Liaoning Provincial Cancer Hospital, Shenyang, China and conducted in compliance with the ethical principles for medical research involving human subjects stated in the World Medical Association Declaration of Helsinki. Informed consent was obtained from all subjects.

### Subjects

A total of 310 FSWs who were detained between October and December, 2013 for alleged engagement in illegal prostitution or sexual acts in public entertainment and leisure facilities (e.g., bars, karaoke rooms, night clubs, beauty spas, salons, saunas, foot and massage parlors) by police in Shenyang, China were identified and approached with the help from of a local police branch. They were all mentally healthy and non-pregnant without structural and anatomic malformations in the reproductive tract and without a history of pelvic irradiation. They had not been screened for cervical neoplasia within five years prior to their police detainment. After exclusion of one subject who refused to undergo gynecological examination, 309 FSWs were finally recruited to the study. As a representative sample of the general population, 1000 women who underwent free annual gynecologic examinations in December, 2013 in the Department of Gynecological Oncology, Liaoning Provincial Cancer Hospital were included for the purpose of normal control. They were employees from a single factory in Shenyany. No specific inclusion and exclusion criteria were applied to the selection of control subjects as a random sample of the general population.

There was no difference in the cultural and racial/ethnic backgrounds between FSWs and control subjects; they were all Han Chinese.

### Papanicolaou (Pap) test and HPV testing

A liquid-based ThinPrep Pap test was performed as previously described [12] and high-risk oncogenic HPV was assessed by the Digene Hybrid Capture II assay (Digene Corporation, Gaithersburg, MD, USA) using a mixture of probes including 16, 18, 31, 33, 35, 39, 45, 51, 52, 56, 58, 59 and 68 as instructed by the manufacturer on the 309 FSWs at the time of their police detainment and on the 1000 healthy control subjects at the time of their regular gynecologic/obstetric visits. Cytological abnormalities revealed by Pap test were classified according to the Bethesda System. When ASCUS or higher grades of squamous intraepithelial lesion were detected, regardless of the HPV testing results (positive or negative), or when HPV was positive, regardless of cytological abnormalities, immediate colposcopy was conducted along with visual inspection with acetic acid (VIA) or Lugol’s iodine (VILI). When colposcopy showed abnormalities, biopsies were taken from transformation zones and acetowhite areas and sent to the Department of Pathology of Liaoning Tumor Hospital for histopathologic assessment. Suspected CIN was graded as mild, moderate or severe based on cytologic findings in the Pap test and/or histologic findings from the colposcopic examination.

### Interview survey

A standard risk factor assessment interview was conducted on 309 FSWs in person at the time of their police detainment. Data on their basic demographics, sexual activities and behaviors (including the age of the first sexual intercourse), reproductive history, menarche, sexually transmitted disease (STD) history, and smoking habit (past and present) were collected.

### Statistical analysis

Data on the prevalence rates of HPV and CIN in the two groups of subjects were analyzed by Pearson’s chi-square test. Putative risk factors for HPV infection were analyzed by univariate and multivariate logistic regression analyses. Differences were considered significant when *P* < 0.05. The statistical software SPSS 11.5 (Chicago, IL, USA) was used.

## Results

### HPV infection and intraepithelial lesions in control subjects and FSWs

Presented in Table 1 are data on HPV infection, cytological abnormalities and cervical intraepithelial cell lesions in FSWS and healthy control subjects assessed. The rate of HPV infection was significantly higher in FSWs than in healthy control subjects (*P* < 0.01). Cytological abnormalities, both ASCUS and ASCUS-H, were detected in a significantly higher proportion in FSWs than in control subjects (*P* < 0.01). In consistence with HPV infection and abnormal cytology, significantly higher occurrence of CIN 1 (*P* < 0.05), CIN 2 (*P* < 0.01) or CIN 3 (*P* < 0.01) was observed in FSWs than in control subjects.

**Table 1:** Differences in HPV infection and cervical intraepithelial lesions between healthy control subjects and FSWs analyzed by Pearson’s chi-square test

	Controls (*n* = 1000)	Sex workers (*n* = 309)
HPV infection	21.00 %	61.90 %**
ASCUS	2.70 %	32.04 %**
ASCUS-H	0.20 %	4.21 %**
CIN1	2.90 %	8.41 %*
CIN2	1.00 %	5.50 %**
CIN3	0.50 %	2.91 %**

Single (*) and double (**) asterisks indicate significant differences from control subjects at *P* < 0.05 and at *P* < 0.01 respectively

### HPV infection in different age groups of female sex workers

To assess the possible age dependency of HPV infection in FSWs, we stratified the 309 female sex workers into 5 different age groups. As shown in Table 2, the prevalence rate of HPV infection ranged from 40.07 to 85.71 %; FSWs either younger than 21 or older than 51 years were at significantly higher risk of HPV infection.

**Table 2:** Age stratification of HPV infection in FSWs

Age (years)	N	HPV prevalence
≤20	21	61.90 %*
21–30	81	41.98 %
31–40	112	41.07 %
41–50	88	52.27 %
>51	7	85.71 %*

Single (*) asterisk indicates a significant difference from control subjects at *P* < 0.05

### Risk factors for HPV infection in FSWs

To identify putative risk factors for HPV infection in FSWs, we first performed univariate regression analysis and the results are presented in Table 3. HPV infection in FSWs was found not significantly associated with marital status, education, smoking, abortion, previous sexual disease, condom use, contraception pills, age of first period, time participating in commercial sex work, vaginal medication use, and regular obstetrical and gynecological examinations. In contrast, young age of first sexual intercourse was the only variable that significantly influenced HPV infection (*P* < 0.001).

**Table 3:** Results of univariate logistic regression analysis of risk factors for HPV infection in FSWs

Category	HPV (+)	HPV (-)	Odds ratio (95 % CI)	P
Marital status				
Single	29	28	1	
Married	55	63	0.84 (0.45–1.59)	0.39
Widowed	7	4	1.69 (0.45–6.41)	0.49
Divorced	54	69	0.76 (0.40–1.42)	0.10
Education				
≤Middle school	110	132	1	
≥High school	35	32	1.31 (0.76–2.26)	0.10
Active smoking				
No	100	110	1	
Yes	45	54	0.92 (0.57–1.48)	0.76
Second hand smoking				
No	76	76	1	
Yes	69	88	0.78 (0.50–1.23)	0.25
Number of abortion				
≤3	130	136	1	
>3	15	28	0.56 (0.29–1.10)	0.11
Sexual disease history				
No	137	148	1	
Yes	8	16	0.54 (0.22–1.30)	0.19
Condom use				
No	29	34	1	
Yes	116	130	1.05 (0.60–1.82)	0.73
Contraception pills				
No	109	121	1	
Yes	36	43	0.93 (0.56–1.55)	0.84
Age of first period (years)				
≤13	28	32	1	0.97
14–15	58	69	0.96 (0.52–1.78)	0.96
16–17	43	43	1.14 (0.59–2.21)	0.77
≥18	16	20	0.91 (0.40–2.10)	0.73
Age of 1st sexual intercourse				
≤20 years	85	72	1	0.00
21–23 years	47	80	0.40 (0.24–0.67)	0.12
≥24 years	13	12	0.99 (0.53–1.85)	0.81
Time as a sex worker				
<1 year	114	127	1	
≥1 year	31	37	0.93 (0.54–1.60)	0.93
Use of vaginal medications				
No	92	102	1	
Yes	53	62	0.95 (0.60–1.50)	0.82
Regular Ob/Gyn exams				
No	95	115	1	
Yes	50	49	1.24 (0.77–1.99)	0.46
Menopause				
No	131	156	1	
Yes	14	8	0.85–5.12	0.11

We next performed multivariate logistic regression analysis. The results clearly revealed that HPV infection in FSWs was significantly associated with the age of first sexual intercourse and post-menopause while the other variables assessed had no effect (Table 4).

**Table 4:** Results of multivariate logistic regression analysis of risk factors HPV infection in FSWs

Variables	Regression coefficient	Odds ratio	95 % CI	*P* value
Marital status	−0.067	0.935	0.727–1.024	0.604
Education	0.445	1.561	0.869–2.805	0.136
Sexual disease history	−0.689	0.502	0.194–1.301	0.156
Smoking	−0.087	0.917	0.550–1.528	0.738
Second hand smoking	−0.279	0.756	0.466–1.227	0.258
Age of first period	−0.004	0.996	0.762–1.301	0.976
Post-Menopause	1.074	2.928	1.099–7.800	0.032
Number of abortion	−0.601	0.548	0.267–1.127	0.102
Condon use	0.093	1.097	0.598–2.012	0.764
Contraception pills	−0.120	0.887	0.497–1.583	0.685
Age of first sex	−0.359	0.699	0.492–0.992	0.045
Time as a sex worker	0.089	1.093	0.600–1.991	0.772

## Discussion

Routine HPV screening for women aged 21 and 65 years can reduce the chance of cervical cancer development [13]. Various HPV testing modalities are available [14]. In this study, we chose the Digene Hybrid Capture 2 High-Risk HPV DNA Test, an approach approved by the US Food and Drug Administration for use in cervical cancer screening, that is highly specific, reproducible, sensitive and reliable [15–17]. With this test, we detected high-risk HPV DNA in 61.90 % of FSWs and in 21.00 % of healthy control subjects (Table 1). It has been documented that there are huge variations in HPV infection in FSWs between different geographic areas and different races. While over 80 % of FSWs were HPV-positive in southern Vietnam [18] and Hungary [19], HPV DNA was detected in 66.8 % of FSWs (*n* = 200) in Peru [20], 57.2 % of FSWs (*n* = 369) in Philippines [21], 49.5 % of FSWs (*n* = 281) in northern Vietnam [22], 47 % of FSWs (*N* = 148) in South Korea [23], 31.6 % of FSWs (*n* = 288) in Australia [24], 22.9 % of FSWs (*n* = 254) in Thailand [25] and 14.4 % of FSWs (*n* = 187) in Singapore [26]. Compared with these worldwide data, the prevalence rate of HPV infection in FSWs detected in this study was in the upper range. Compared with reports from other Chinese studies, the HPV infection rate in FSWs in Shenyang was very close to that in FSWs in Huzhou (66.7 %) [11], but was much higher than that in FSWs in Guangxi (38.9 %) [8]. Currently, the exact reasons underlying the reported difference in HPV prevalence in FSWs in different areas in China are unknown and further studies are warranted.

In consistency with HPV infection, cervical dysplasia, assessed either histologically or cytologically, was significantly more prevalent in FSWs than in healthy controls in this study (Table 1). In the current literature, data on cervical epithelial cell lesions in FSWs are scarce. In a study involving 90 FSWs aged 18 to 58 years in Antananarivo, Madagascar, the prevalence was 3.3 % for low-grade squamous intraepithelial lesions and 18.9 % for ASCUS while no high-grade lesion was detected [27]. In another study from Hong Kang, CIN 1–3 lesions were observed in 9.8 % of 235 FSWs [28]. Comparatively, squamous intraepithelial lesions, particularly ASC-US (32.04 %) and low-grade lesions, detected in this study were all higher than those reported in the two studies mentioned above. It has been well documented that free-of-charge screening services to FSWs is very helpful not only in early detection and proper follow-up in case of abnormal Pap tests but also in increasing the awareness of women’s health issues [29]. Given the substantially high incidence of ASC-US and low-grade lesions observed in this study, a free or practically feasible and cost-efficient HPV screening program is urgently needed to minimize the risk for cervical cancer in FSWs in Shenyang.

Numerous factors have been assessed for a putative link with HPV infection in FSWs in previous studies. However, inconsistent results have been obtained. In this study, we assessed various socio-demographic and behavioral variables, among which age of first sexual intercourse and menopause were the only two risk factors for HPV infection in FSWs in Shenyang.

In agreement with our findings, young age (OR 0.699, 95 % CI 0.492–0.992) and menopause (OR 2.928, 95 % CI 1.099–7.800) have been suggested as independent risk factors for HPV infection in FSWs in previous studies [8, 10, 22, 24, 30]. The exact mechanisms underlying increased vulnerability to HPV infection in young and post-menopausal FSWs are not fully understood. However, inadequate acquired immunity at young age and decreased acquired immunity at post-menopausal age might be one of the explanations [30]. Supporting this speculation, Sivro and colleagues have demonstrated that there are significant hormone-associated changes in systemic and mucosal cytokine/chemokine production, which may have implications for the age-related decline in the ability to fight against infections in young and post-menopausal women [31].

Education levels [10, 18] and years of commercial sex work [10] have been demonstrated to affect HPV infection in FSWs in other studies. Inconsistent with these studies, the present study showed that HPV infection in FSWs in Shenyang was not associated with education levels or years of commercial sex work.

The impact of cigarette smoking on HPV prevalence has been assessed in both men and women [32]. However, in the current literature, little information is available regarding the difference in HPV infection between smoking and non-smoking FSWs, although it has been reported that the risk for FSWs to develop preinvasive or invasive cervical lesions increase with the number of cigarettes they smoked per day and their years of smoking [33]. In this study, the effect of smoking, either active or passive, on HPV infection was not demonstrated in FSWs in Shenyang and warrants further assessment in the future.

The benefit of condom use in protecting HPV infection in women is debatable [34]. While a role for condom use in reducing HPV infection in FSWs has been reported [11, 27, 30], there is an argument that condoms may not completely cover the infected areas, thus rendering insufficient protection against HPV infection [19]. In this study, no significant difference was found in HPV prevalence between condom users and non-condom users, supporting a limited capacity of condoms in HPV protection.

In this study, HPV infection in FSWs in Shenyang was associated with all other socio-demographic, medical and behavioral variables including marital status, number of abortions, sexual disease history, use of contraception pills and other medications, age of puberty, and previous gynecologic and obstetric examinations. Nevertheless, these findings need to be further validated in studies with a large sample size.

This study has several limitations. First, ThinPap test and cytological evaluation were performed only once on both FSWs and control subjects. This cannot totally rule out the possibility of miss the true abnormalities. Second, in evaluating the usefulness of condom use in protecting against HPV infection, only the information on ‘current condom use’ at the time of interview was collected. This might not represent a complete picture. A more accurate answer would have been obtained if condom use had been stratified into three scenarios: no condom use either in the past or at the present; condom use always; and condom use sometimes.

## Conclusions

HPV prevalence and cervical lesion incidence were substantially higher in FSWs than in healthy control subjects in Shenyang. Falling in the upper range of the reported values across different countries, HPV prevalence detected in FSWs in this study was similar to that for FSWs in southeast Huzhou but higher than that for FSWs in southwest Guangxi within China. Age of first sexual intercourse and menopause were two independent risk factors for HPV infection in our sample of FSWs. Public health endeavor is urgently needed to reduce the risk of HPV infection in FSWs in the northeast Chinese city of Shenyang.
